# Electroacupuncture at Zusanli (ST36) Repairs Interstitial Cells of Cajal and Upregulates c-Kit Expression in Rats with SCI-Induced Neurogenic Bowel Dysfunction

**DOI:** 10.1155/2020/8896123

**Published:** 2020-11-27

**Authors:** Yujie Yang, Jie Cheng, Yongni Zhang, Jiabao Guo, Bin Xie, Wenyi Zhang, Zhaojin Zhu, Yi Zhu

**Affiliations:** ^1^Department of Biomedical Sciences, City University of Hong Kong, Kowloon, Hong Kong; ^2^The Second Clinical Medical School, Nanjing University of Chinese Medicine, Nanjing, Jiangsu, China; ^3^Yueyang Hospital of Integrated Traditional Chinese and Western Medicine, Shanghai, China; ^4^Department of Rehabilitation Medicine, The Second School of Clinical Medicine, Xuzhou Medical University, Xuzhou, Jiangsu, China; ^5^Jiangyin Orthopedics Hospital of Traditional Chinese Medicine, Wuxi, Jiangsu, China; ^6^Zhongshan Rehabilitation Branch of Jiangsu Provincial Hospital, Nanjing, Jiangsu, China; ^7^Department of Rehabilitation, Changzhou Higher Vocational and Technical College of Health, Changzhou, Jiangsu, China; ^8^Department of Pain and Musculoskeletal Rehabilitation, The Fifth Affiliated Hospital of Zhengzhou University, Zhengzhou, Henan, China

## Abstract

**Background:**

Electroacupuncture (EA) could improve colonic transit activity in rats with neurogenic bowel dysfunction (NBD) caused by spinal cord injury (SCI). The function of interstitial cells of Cajal (ICCs) and c-Kit expression may play essential roles in this process. *Material and Methods*. Thirty-six Sprague Dawley rats were randomized to the sham group, the SCI group, or the SCI + EA group (bilateral Zusanli, 30 min/day, 14 days). Changes in the ultrastructural morphology of ICCs were observed. The c-Kit expression on different levels was analyzed by immunohistochemistry, Western blotting, and RT-qPCR, respectively.

**Results:**

Abnormal morphology of ICCs and downregulation of the c-Kit expression occurred after SCI. While the number of ICCs was increased, the ultrastructural morphology was improved significantly in EA rats. They also showed better improvement in c-Kit expression at both protein and gene levels.

**Conclusion:**

Abnormal ICCs in colon tissues and the downregulated expression of c-Kit could be observed after SCI. EA at Zusanli (ST36) could improve the colon function by repairing the morphology and increasing the number of ICCs and upregulating c-Kit expression.

## 1. Introduction

Neurogenic bowel dysfunction (NBD) is a term that refers to colonic dysfunction caused by the central nervous system (CNS) disease or injury [1]. It occurs in almost all patients with a chronic spinal cord injury (SCI) [2]. They suffer from functional obstruction, constipation, fecal incontinence, abdominal pain, or their combination [3]. More than 40% of SCI patients complained that the NBD seriously affects their quality of life [4, 5] because it not only aggravates the physical condition but also causes the psychological problem by imposing restrictions on their participation in daily life activities and threatening their privacy and dignity [4, 5].

Thus, management of bowel function ranks in the most priorities among SCI patients [1]. Various therapeutic interventions have been applied for NBD management [3], including multiple forms of electrical stimulation [6]. EA, as a convenient, repeatable, and less-lesion intervention, is commonly used to treat in both clinical trials and laboratory experiments [7]. The combination of traditional Chinese medicine theory and modern neuromodulation theory make EA more acceptable than the traditional acupuncture. Zusanli (ST36) is one of the most frequently used acupoints to prevent and treat gastrointestinal disorders. Although a considerable amount of research has reported the effect and mechanism of EA on gastrointestinal dysfunction [8–12] or other complications of SCI (such as the neurogenic bladder dysfunction) [13–15], the quality and quantity of EA studies on SCI-induced NBD are relatively insufficient. Some research supported the effect of EA on NBD patients or animal models. A clinical trial has proven that EA is effective at managing NBD by decreasing the burden of bowel care and episodes of fecal incontinence in SCI patients [16]. Our previous study has found that EA at ST36 could increase the colonic propulsive movement in SCI rats [17, 18]. However, the mechanism of EA on NBD remains unclear.

The decline in colonic motility and delay in colonic transit are considered as the primary mechanisms of NBD after SCI [19]. Interstitial cells of Cajal (ICCs) have a function in regulating gastrointestinal motility by generating and propagating slow waves as well as transducing signals between the enteric nervous system (ENS) and smooth muscle cells [20]. Studies have shown that a reduction in the ICC population may cause slow-transit constipation [21, 22]. A clinical histology study conducted in the Netherlands confirmed that patients with spina bifida or SCI experienced a loss of ICCs and neurons in the myenteric plexus, compared with control patients without gastrointestinal motility disorders [23]. The proto-oncogene c-kit expressed by ICCs encodes the tyrosine kinase receptor (c-Kit). Generally, the c-Kit is considered as the identification marker of ICCs [24]. Some studies found that c-Kit is closely associated with the development, differentiation, and functional maintenance of ICCs in the intestine [25, 26]. In addition, several gastrointestinal motility disorders have been linked to depletion of c-Kit-positive ICCs [27, 28], and re-expression of c-kit protein may contribute to the functional recovery of ICCs [29]. Studies have reported that EA at Zusanli can promote colonic motility [30, 31] and increase the expression of ICCs in partial intestinal obstruction [32]. However, the underlying mechanism has not yet been investigated. Thus, this study aimed to explore the possible mechanisms of EA therapy on defecation dysfunction after SCI.

## 2. Material and Methods

### 2.1. Animals and Grouping

Thirty-six female adult-specific pathogen-free Sprague Dawley rats adopted from the Sino-British Sippr/BK Lab Animal Co. Ltd. (Shanghai, China) were used in this research. All the animals were raised under standardized laboratory conditions with a light-dark cycle (12 : 12). The ambient temperature was kept between 21 and 25°C, and the relative humidity was set to 50–55%. They were fed adaptively for ten days with free access to water and food until reaching a weight of 300 ± 20 g.

Twenty-four rats were selected randomly for the SCI model establishment, and the remaining twelve rats received the sham operation. Only the successful SCI models were included in further experiments and then randomly assigned to either the SCI group or the SCI + EA group. Animals that received sham surgery were included in the sham group as a control.

All the experiments were performed in the Jiangsu Province Key Laboratory of Acupuncture and were approved by the Center for Safety Evaluation of Research in the Nanjing University of Chinese Medicine.

### 2.2. SCI Modeling and Sham Surgery

The SCI model in rats was established with the weight-drop method. The atropine sulfate (0.05 mg/kg, s.c.) was used to inhibit tracheal secretions. Fifteen minutes later, anesthesia was induced with pentobarbital sodium (50 mg/kg, i.p.). Animals then were mounted on the operating table in a prone position. A middle dorsal incision was performed from T10 to T13 following skin preparation. The vertebral plates and spinous process of T11 and T12 were exposed and then removed by laminectomy to make a window revealing the spinal cord. The spinal cord was contused using the New York University Impactor I, which could provide a drop of 10-gram rob from a height of 60 mm. A subdural hemorrhage followed by twitching of posterior limbs and lashing of tails could be observed immediately after the wound. The operative incisions were sutured after debridement.

For animals that received sham operation, the operative incisions were sutured following the removal of the spinous process and vertebral plates from T11 to T12 without any injury on the spinal cord.

All animals were accessed by the modified Basso–Beattie–Bresnahan locomotor scale (mBBB, Table S2) [33] before and 24 h after the operation for judging and evaluating whether or not the modeling succeeded. Each time the assessment was performed by two assessors independently. All animals got 21 points before any operation was performed. Twenty-four hours after the SCI modeling surgery, rats that got 0 points will be considered as successful models. Animals that received sham surgery were included in the further experiments only if they got 21 points 24 h postoperatively.

### 2.3. Postoperative Nursing

All the rats received intraperitoneal injections of gentamicin (5000 U/kg) daily from the operating day. The abdomen, perineum, and hind limbs of the SCI rats were cleaned every 12 h after Crede's maneuver that was performed to assist with urination. The passive motion was performed on the hind limbs every day before the intervention.

### 2.4. Electroacupuncture (EA)

EA intervention started from the day after the surgery once the rats were considered as the successful model. Animals in the SCI + EA group were mounted on an operation platform in a supine position on the treatment table. The acupoints selected for intervention are the bilateral Zusanli points (ST36), which are 5 mm below the fibular head and 1 mm lateral to the anterior border of tibia [34]. After local disinfection with iodophor, the acupuncture needles (Hua Tuo, 0.25*∗*13 mm; Suzhou Medical Appliance Factory, Co. Ltd., Jiangsu, China) were perpendicularly inserted to a depth of 5 mm. Then, the needles were connected with a Hua Tuo electroacupuncture apparatus (SDZ-II; Suzhou Medical Appliance Factory, Co. Ltd., Jiangsu, China) with the disperse-dense wave at a current intensity of 1-2 mA and a frequency of 3 Hz/15 Hz. The stimulation strength was limited to a tolerable range within which rats could freely vocalize, and the subtle vibration of the needle could be observed. At the same time, animals of the sham group and the SCI groups were mounted in the same method only without EA. All the interventions started around 11 am and lasted 30 min, once daily, for 14 days.

### 2.5. Tissue Preparation

After a 2-week intervention, rats fasted for 24 h were sacrificed by cervical dislocation under deep anesthesia. One centimeter sections of the proximal colon (one centimeter below the cecum) were removed from all animals for subsequent examinations. For the ultrastructural morphology, the proximal colon tissues were immersed in 5% glutaraldehyde at 4°C for 2 h after removing the mucosae and then postfixed in 1% osmic acid at 4°C for 2 h before the 2% uranyl acetate staining. After dehydration with a graded series of ethanol at 4°C, the specimens were stained with toluidine blue. Then, they were sliced to semithin sections with a 70–80 nm thickness using an ultramicrotome (Leica RM2145, Germany). For the immunohistochemistry analysis, 5 *μ*m-thick slices of the proximal colon tissues were cut from the 4% paraformaldehyde-fixed, paraffin wax embedded blocks for further processing. For the RT-qPCR analysis and the Western blotting, the proximal colon tissues with dissection of the mucosa were immersed in the liquid nitrogen and then stored at −80°C.

### 2.6. Ultrastructural Morphology

The transmission electron microscopy (TEM) was applied to observe the ultrastructural changes in colonic ICCs. The tissue specimens were observed under a transmission electron microscope (JEM-1010; JEOL Ltd., Japan) at 4000x and 25000x magnification, respectively. The images of the ultrastructure of ICCs were searched and captured by researchers who were blinded to the grouping.

### 2.7. Immunohistochemistry (IHC)

The IHC was performed to detect the c-Kit-positive ICCs in the proximal colon tissues. The paraffin slices of the proximal colon tissues were placed in a 60°C oven for 2 h. Then, they were dewaxed with xylene and rehydrated through a graded series of ethanol. Antigen was retrieved in the boiled citric acid buffer after the activity of endogenous peroxidase was blocked with 0.30% H_2_O_2_. Then, the sample slices were blocked with 5% bovine serum albumin for one hour at room temperature. Slices were incubated with rabbit anti-rat c-Kit multiclonal antibody (1 : 100; Santa Cruz Biotechnology, Inc., USA) at 4°C overnight. After they were washed three times in the phosphate-buffered saline (PBS), the sample slices were incubated with the secondary antibody (1 : 500; SABC kit-SA1022; BOSTER Biological Technology Co. Ltd., Wuhan, China) and later with the streptavidin-biotin Complex (SABC) system. Afterward, the sample slices were washed in PBS after being incubated with 3,3-diaminobenzidine (DAB). Finally, the sample slices were counterstained with hematoxylin. Staining specimens were imaged using an optical microscope (Olympus, Japan) at 400 magnification, and the c-Kit-positive ICCs were quantified by detecting ten randomly selected high power fields from each slice. The average optical density (AOD) of positively stained areas, which is used to measure the staining intensity, was analyzed by the JD801 morphological microimage analysis system (Nanjing Jieda Company, Jiangsu, China).

### 2.8. Western Blotting (WB)

The WB was used to analyze the expression level of colonic c-Kit protein. Frozen samples of proximal colon tissues were homogenized in the RIPA lysis buffer (Nanjing Vazyme Biotech Co. Ltd., Jiangsu, China) with a handheld homogenizer. The homogenate was centrifuged at 10000×*g* at 4°C for 5 min. Only the supernatant was collected for further use. The concentration of protein in the supernatant was quantified by the bicinchoninic acid (BCA) method. Next, the protein samples were boiled in a sodium dodecyl sulfate (SDS) loading buffer for 5 min. Then, the samples were loaded onto an 8% polyacrylamide gel for electrophoresis. After the proteins were separated, they were transferred onto a polyvinylidene fluoride (PVDF) membrane that would be incubated with 5% nonfat milk at room temperature for 2 h to block nonspecific binding sites. Afterward, the membranes were incubated with the anti-c-Kit antibodies (1 : 200; Santa Cruz Biotechnology, Inc., USA) and tubulin (1 : 5000; Nanjing Vazyme Biotech Co. Ltd., Jiangsu, China) at 4°C overnight on a rotating platform. Next, the PVDF membranes were washed with PBS four times before and after the incubation with a secondary antibody (1 : 5000; one hour at room temperature). A chemiluminescence imaging system (ChemiScope3500; ClinX Science Instruments Co. Ltd., Shanghai, China) was used to scan the immunoblots (Figure S1). The gray value of immunoreactive protein bands was normalized to tubulin expression and was analyzed by a chemical analysis system (Gel Analysis V2.02; Table S1).

### 2.9. RT-qPCR

The RT-qPCR was applied to measure the expression level of colonic c-Kit protein mRNA. The total RNA was isolated from the lysed proximal colon tissues by using an RNA Isolator Total RNA Extraction Reagent (R401-01; Nanjing Vazyme Biotech Co. Ltd., Jiangsu, China). RNA concentration and integrity were analyzed by measuring ultraviolet absorbance ratios at 260 nm/280 nm. The total RNA was reverse-transcribed to cDNA with the HiScriptQ RT SuperMix (R213; Nanjing Vazyme Biotech Co. Ltd., Jiangsu, China). The AceQ® qPCR SYBR Green Master Mix (Nanjing Vazyme Biotech Co. Ltd., Jiangsu, China) was used for the RT-qPCR of cDNA. The c-Kit primers (forward: CCTCGCCTCCAAGAACTGTATT; reverse: GCCGTGCATTTCCTTTTACC) were designed and synthesized by Vazyme Biotech Co. Ltd. (Nanjing, China). Glyceraldehyde-3-phosphate dehydrogenase (GAPDH) was treated as an internal control (forward: GAGTCCACTGGCGTCTTCA; reverse: GGGGTGCTAAGCAGTTGGT). The StepOne Plus system (Applied Biosystems, USA) was used for the amplification reaction, and the conditions of thermal cycling were 95°C for 5 min in the start cycle, 95°C for 10 s, and 60°C for 30 s in 40 subsequent cycles. Data were analyzed with the 2^−ΔΔ*Ct*^ methods.

### 2.10. Statistical Analysis

SPSS 20.0 (IBM, USA) was used to do all the statistical analysis. Data were reported as means ± standard deviation (s.d.). The one-way ANOVA was performed to analyze the difference among groups. For the pairwise comparisons, the LSD test will be used when the variance is homoscedastic, and the Dunnett T3 test will be applied when the variation is heteroscedastic. Statistical significance (*p*-value) was set smaller than 0.05.

## 3. Results

### 3.1. Establishment of the Rat Model of SCI

According to the mBBB scale, all rats scored 21 points before any surgical procedure. Twenty-four out of 36 rats received the modeling surgery by the modified drop weight method, and two of them died during the modeling operation. The remaining 22 rats, which scored 0 in the BBB scale twenty-four hours postoperatively, were considered as successfully established SCI models and then were randomly assigned either to the SCI + EA group or to the SCI group. Twelve rats received the sham surgery, and 2 of them were excluded because they had a decreased mBBB score, which indicated partial injury of the spinal cord. The fecal characteristics and defecation condition of different groups were described previously [17, 18].

### 3.2. Morphology of ICCs Repaired by EA

**Figure 1:**
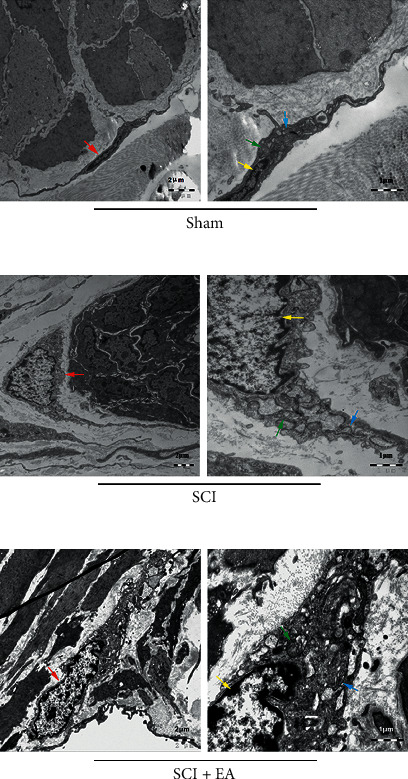
Morphology of ICCs in the colon tissues from rats in different groups. (a) In the sham group (*n* = 5), the typical shape of the cell body and nucleus was observed, and the intracellular organelles were abundant and healthy. (b) In the SCI group (*n* = 5), the structural abnormalities of ICCs and its organelles were observed, and vacuoles and organelle decreasing happened in the cytoplasm. (c) In the SCI + EA group (*n* = 5), the shape of the cell body and intracellular structures were closer to those in the sham group, although the cytoplasm still contained some vacuoles. Red arrow, interstitial cells of Cajal (ICC); yellow arrow, nucleus; green arrow, mitochondria; blue arrow, endoplasmic reticulum. Scale bar, 2 *μ*m in the left panels and 1 *μ*m in the right panels.

As observed under the transmission electron microscope, the cell body of colonic ICCs was typically fusiform-shaped with an elongated nucleus in the sham group (Figure 1(a), left panel). The condensed chromatin was scattered throughout the nucleus, surrounded by the extended cytoplasm. The cytoplasm contained abundant mitochondria, ribosomes, endoplasmic reticulum, and Golgi apparatus (Figure 1(a), right panel).

The morphological damage of ICCs in the colon occurred after the SCI. The shape of cell bodies and the nucleus were irregular (Figure 1(b), left panel). Vacuoles, organelle decrease, and structural abnormalities were observed inside the cytoplasm. The mitochondria were swollen, dissolved, or even ruptured and vacuolated. Lipid droplets were observed in the dilated and degranulated endoplasmic reticulum (Figure 1(b), right panel).

However, we found that the morphological lesion of colonic ICCs was repaired after the EA treatment. It seems that the shape of colonic ICCs' cell bodies and nucleus were more similar to those in the sham group (Figure 1(c), left panel). Although there were some vacuoles contained in the cytoplasm, the structure of the organelle was relatively complete. The mitochondria swelled slightly, and the endoplasmic reticulum expanded (Figure 1(c), right panel). Overall, the above findings indicate that EA could rescue the morphology of colonic ICCs after SCI.

### 3.3. c-Kit Immunoreactive ICC Number Increased by EA

**Figure 2:**
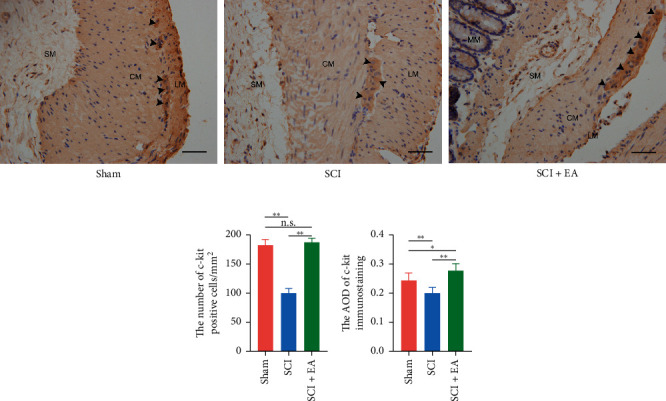
Immunochemistry of ICCs in the colon tissues from rats in different groups. (a) Representative images of c-Kit immunoreactive ICCs. The mass of c-Kit-positive ICCs in the SCI group (*n* = 5) was significantly lower than the sham (*n* = 5) and SCI + EA (*n* = 5) groups. Scale bar, 100 *μ*m. (b) The quantitative analysis of c-Kit-positive cell number. (c) The quantitative analysis of the average optical density (AOD). Each bar is shown as mean ± s.d. (n.s., no significance; ^*∗*^*p* < 0.05; ^*∗∗*^*p* < 0.01).

As shown in Figure 2(a), there were apparent differences in the density of c-Kit immunoreactive ICCs among three groups. In contrast with the sham group, the mass of c-Kit-positive ICCs decreased significantly after the SCI. While after the EA treatment, the expression of colonic c-Kit rebounded to the same level in the sham group. The quantitative analysis of c-Kit-positive cell number and the AOD further confirmed the above results (Figures 2(b) and 2(c)). The SCI group showed a fewer number of c-Kit immunoreactive cells (100.38 ± 7.75 vs. 182.92 ± 9.74, *p* < 0.01) and a lower AOD (0.20 ± 0.02 vs. 0.24 ± 0.02, *p* < 0.01) than the sham group. However, the EA treatment reversed the decline caused by the SCI. The number of c-Kit immunoreactive cells (187.702 ± 6.76 vs. 100.38 ± 7.75, *p* < 0.01) and the AOD (0.28 ± 0.02 vs.0.20 ± 0.02) were significantly increased in the SCI + EA group.

### 3.4. c-Kit mRNA and Protein Expression Level Increased by EA

**Figure 3:**
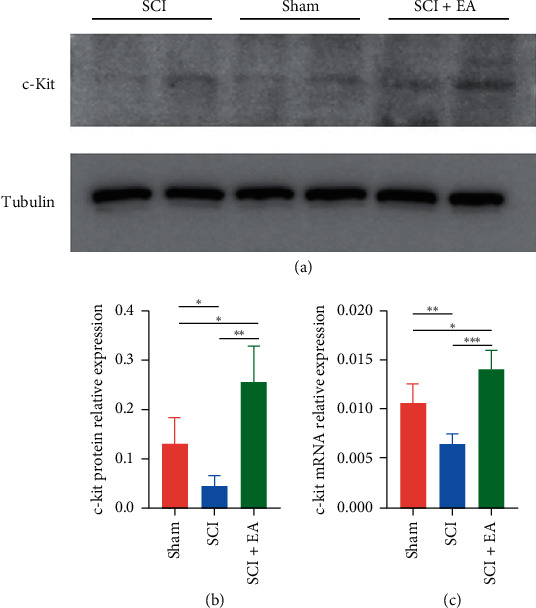
The c-Kit mRNA and protein expression in the colon tissues from rats in different groups. (a) Western blotting assays of the c-Kit protein expression from different groups (*n* = 4, each group). (b) The relative expression of the c-Kit protein (normalized with the Tubulin) (*n* = 4, each group). (c) The relative expression of c-Kit mRNA (normalized with the tubulin) (*n* = 4, each group). Each bar is shown as mean ± s.d. (^*∗*^*p* < 0.05; ^*∗∗*^*p* < 0.01).

The results of the colonic c-Kit mRNA and protein expression confirmed what we found in the immunohistochemistry. The expression level of c-Kit protein (Figures 3(a) and 3(b)) and mRNA (Figure 3(c)) in colon tissues varied among groups. The mRNA expression and protein levels of c-Kit in the SCI rats were significantly lower than the sham rats (protein: 0.04 ± 0.02 vs. 0.13 ± 0.05, *p* < 0.01; mRNA: 0.006 ± 0.001 vs. 0.010 ± 0.002, *p* < 0.01). After the EA treatment, the mRNA expression and protein levels of c-Kit in the colon tissues increased significantly compared with the SCI rats (protein: 0.25 ± 0.07 vs. 0.04 ± 0.02, *p* < 0.01; mRNA: 0.014 ± 0.002 vs. 0.006 ± 0.001, *p* < 0.01).

## 4. Discussion

Our previous study found that EA could improve the bowel function in rats with SCI-induced NBD and revealed the partial underlying mechanism behind the intervention [17, 18]. We found that EA could improve the fecal characteristics and shorten the defecating time of SCI rats. The studies also showed that EA could regulate circadian rhythmicity of intestinal motility and downregulate the expression of neuronal nitric oxide synthase in colon tissues. In this research, we explored the potential mechanism of EA from the aspect of its effect on the ICCs. Results revealed that EA could improve the colon function after SCI by repairing morphology and the number of ICCs and upregulating c-Kit expression.

ICCs are one of the various interstitial cell types contained in gastrointestinal smooth muscles. Under physiological conditions, ICCs were rich in mitochondria; the moderately well developed inner membrane system included the Golgi complex and endoplasmic reticulum. ICCs play an essential role in driving and maintaining the normal functions of gastrointestinal smooth muscles, and the loss or remodeling of ICCs could contribute to intestinal motility dysfunction [35]. As the pacemaker cells, ICCs could generate and conduct slow electrical waves, which are the fundamental of the segmental contraction and peristalsis of the digestive tract [20]. Studies found that slow-wave activity recorded from wild-type and heterozygote mice could not be detected from the W/WV mutated mice that lacked ICCs in the small intestinal muscles [36, 37]. Similar results exist in the Ws/Ws mutated rats. ICCs in the colonic muscles of the Ws/Ws mutated rats were incompletely lost, and the electrical activity shows an irregular pattern and lower frequency than what occurred in the wild-type animals [38]. ICCs offer a pathway for the propagation of slow waves for the smooth muscle cells that could not regenerate the slow waves on their own. Some research found that the slow waves that occurred in the healthy colonic tissue could not propagate to the area in which the ICCs were dysfunctional [39, 40]. As described in our previous studies [17, 18], rats experienced a T11-12 level SCI showed exhibited dry and hard stool, a decrease of fecal weight, and prolonged defecation, which was the result of the colonic transit disorder. The colonic transmission function could be improved by EA treatment later. Our experiments showed that the above changes in colonic function were associated with the ICCs. The ultrastructure injuries in ICCs could be observed in animals with SCI, and later those injuries were repaired after the EA treatment.

Except as an identification marker of ICCs, c-Kit plays an vital role in the ICC function. The importance of c-Kit and its ligand emerged from the mutated animals such as W/WV mice, Ws/Ws rats, and Sl/Sld mice. In those animals, reduction in the ICC population and loss of pacemaker activity were observed [37, 41, 42]. The similar results could be found in the animals treated with c-Kit antibodies [24, 40, 43]. In this study, we also found the downregulation of c-Kit expression in colon tissues of SCI rats and the rescuing effect of EA.

The intestinal function is double-innervated by the CNS and ENS. Although the ENS could regulate intestinal motility when its connection with the CNS is completely cut off [44], the activity of ENS declined due to loss of modulation from the CNS via sympathetic and parasympathetic nerves [45]. For instance, our previous study showed that the concentration of nitric oxide synthase (nNOS), which could synthesize nitric oxide served as primary neurotransmitters for inhibitory motor neurons, significantly increased in the colon of SCI rats [17]. Substantial evidence suggests enteric motor neurons directly innervate ICCs. The dysfunction of neurotransmitter release could lead to the morphological change and functional disorder of ICCs [45]. ICCs could facilitate communication between enteric motor neurons and smooth muscle cells by transducing the excitatory and inhibitory inputs. Research studies found that the loss of ICCs resulted in the reduction of excitatory and inhibitory potentials in smooth muscles of W/WV mice under circumstances in which the functions of enteric motor neurons and smooth muscle cells themselves are normal [46, 47]. On the other hand, many studies have worked on the mechanism of how EA could regulate gastrointestinal function. Results showed that EA could modulate the excitation and inhibition of the ENS [48], and the high-frequency EA at acupoint ST36 could induce the regeneration of enteric neurons [49]. In our previous study, EA at ST36 could downregulate the nNOS expression in the rats' colon [17]. It was also found that EA is capable of affecting the gastrointestinal motility by regulating the activity of extrinsic autonomic nerves (enhancing vagal activity and suppressing sympathetic activity) [50–52].

Based on these studies so far, there is one probable assumption about the mechanism of SCI-induced NBD and EA treatment. After SCI, the activity of ENS decreased, sending fewer signals to ICCs and then resulting in the ultrastructural damage and dysfunction of ICCs, eventually leading to degradation of the motility of colonic smooth muscles. EA activates the peripheral nerves, transmitting signals to parasympathetic nerves in the sacral segments, facilitating the activity of ENS. The ENS activity contributes to the functional recovery of ICCs innervated by enteric motor neurons, finally causing the retrieval of colonic transit function. More experiments are needed to confirm the proposed mechanism.

## 5. Conclusion

Abnormal ICCs and the downregulated expression of c-Kit could be observed after SCI, which could be one of the causes of NBD. EA at Zusanli (ST36) could improve the colon function after SCI, and its effect on repairing the morphology and number of ICCs and upregulating c-Kit expression could be one of the potential mechanisms. Further research is necessary to reveal the deep mechanism behind the dysfunction and intervention.

## Data Availability

All the data used to support the findings of this study are available from the corresponding author upon request.

## Supplementary Materials

Supplementary Materials Original data for the Western blot (Figure S1, Table S1) and the modified Basso–Beattie–Bresnahan locomotor scale (Table S2).
