# Supplementary material for: Electroacupuncture at Zusanli (ST36) Repairs Interstitial Cells of Cajal and Upregulates c-Kit Expression in Rats with SCI-Induced Neurogenic Bowel Dysfunction
Source: Evid Based Complement Alternat Med. 2020 Nov 27;2020:8896123. doi: 10.1155/2020/8896123 (PMC7718052; doi:10.1155/2020/8896123)
Supplement: Supplementary Materials — Original data for the Western blot (Figure S1, Table S1) and the modified Basso–Beattie–Bresnahan locomotor scale (Table S2). [file 8896123.f1.docx]

Supplementary Materials for

**Electroacupuncture at Zusanli (ST36) Repairs Interstitial Cells of Cajal and Up-regulate c-Kit Expression in Rats with SCI-induced Neurogenic Bowel Dysfunction**

Yujie Yang, Jie Cheng, Yongni Zhang , Jiabao Guo, Bin Xie , Wenyi Zhang , Zhaojin Zhu^7^, Yi Zhu*

* Corresponding Author Email: [zhuyi1010@163.com](mailto:zhuyi1010@163.com)

**This file includes:**

Original data for the Western Blot (Fig. S1, Table S1)

The modified Basso-Beattie-Bresnahan locomotor scale (Table S2)


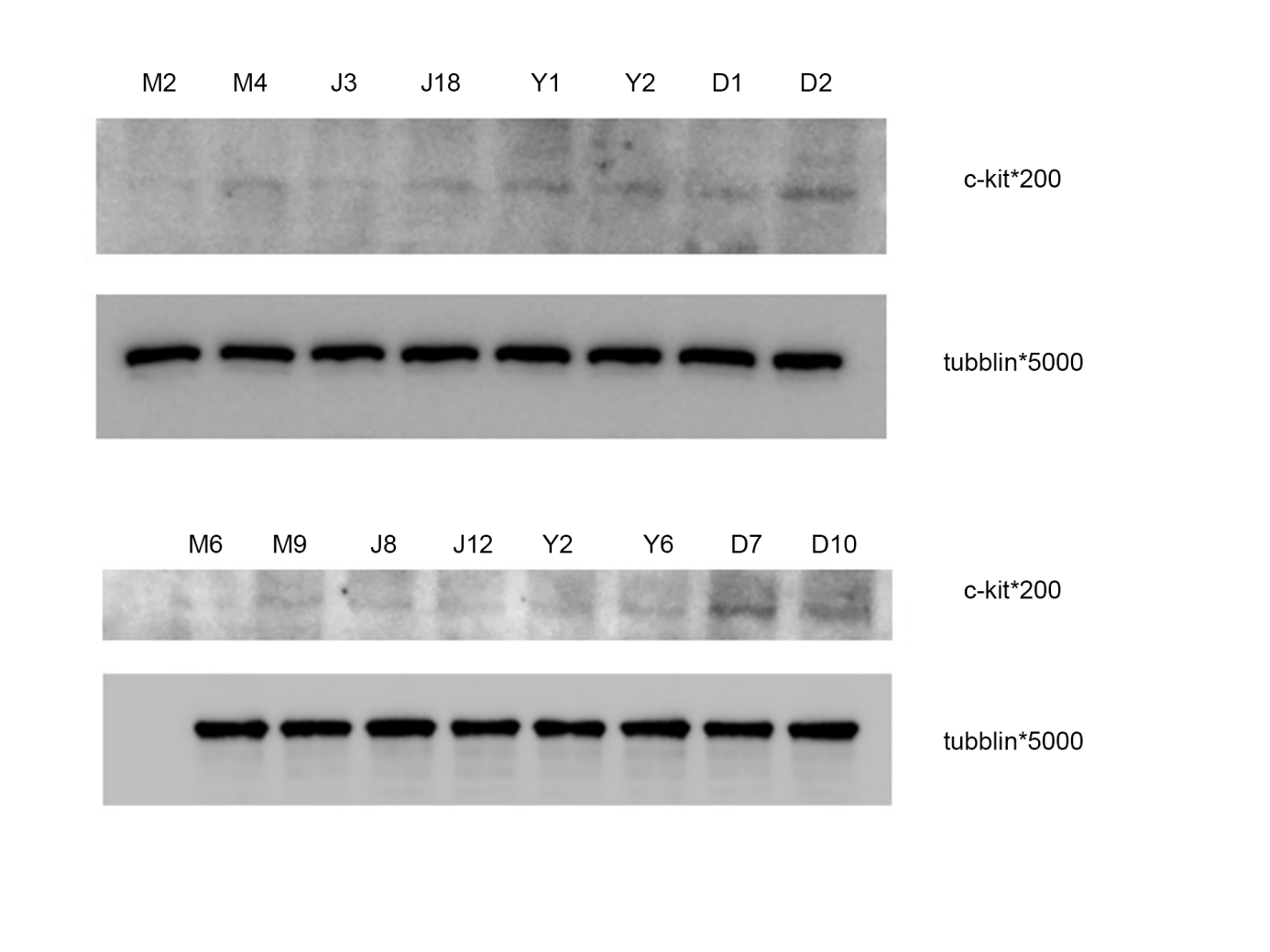


**Figure S1.The Western Blot bands.** M: the SCI group, J: the Shamgroup, D: the SCI+EA group,Y are not involved in this experiment

**Table S1. The grey value of immunoreactive protein bands**

| **TUBLIN** | **M2** | **M4** | **M6** | **M9** | **J3** | **J18** | **J8** | **J12** | **Y5** | **Y9** | **Y2** | **Y6** | **D1** | **D9** | **D7** | **D10** |
| --- | --- | --- | --- | --- | --- | --- | --- | --- | --- | --- | --- | --- | --- | --- | --- | --- |
|  | Lane 1 | Lane 2 | Lane 1 | Lane 2 | Lane 3 | Lane 4 | Lane 3 | Lane 4 | Lane 5 | Lane 6 | Lane 5 | Lane 6 | Lane 7 | Lane 8 | Lane 7 | Lane 8 |
| Band 1（gary value） | 923652 | 919128 | 792124 | 768692 | 801540 | 849108 | 729164 | 692800 | 906108 | 913212 | 723336 | 768520 | 917552 | 825888 | 750536 | 761188 |
|  |  |  |  |  |  |  |  |  |  |  |  |  |  |  |  |  |
| **Ckit** | **M2** | **M4** | **M6** | **M9** | **J3** | **J18** | **J8** | **J12** | **Y5** | **Y9** | **Y2** | **Y6** | **D1** | **D9** | **D7** | **D10** |
|  | Lane 1 | Lane 2 | Lane 1 | Lane 2 | Lane 3 | Lane 4 | Lane 3 | Lane 4 | Lane 5 | Lane 6 | Lane 5 | Lane 6 | Lane 7 | Lane 8 | Lane 7 | Lane 8 |
| Band 1（gary value） | 25491 | 50643 | 21563 | 53165 | 104349 | 174163 | 69843 | 62966 | 166863 | 188156 | 50032 | 53169 | 271294 | 279132 | 151183 | 139862 |
|  |  |  |  |  |  |  |  |  |  |  |  |  |  |  |  |  |
|  | **M2** | **M4** | **M6** | **M9** | **J3** | **J18** | **J8** | **J12** | **Y5** | **Y9** | **Y2** | **Y6** | **D1** | **D9** | **D7** | **D10** |
| **Ckit /TUBLIN** | 0.027598 | 0.055099 | 0.027222 | 0.069163 | 0.130186 | 0.205113 | 0.095785 | 0.090886 | 0.184154 | 0.206038 | 0.069168 | 0.069184 | 0.295672 | 0.337978 | 0.201433 | 0.183742 |

M: the SCI group, J: the Shamgroup, D: the SCI+EA group,Y are not involved in this experiment

**Table S2. The modified Basso-Beattie-Bresnahan locomotor scale (mBBB)**

| 0 | No observable hindlimb (HL) movement |
| --- | --- |
| 1 | Slight movement of one or two joints, usually the hip and/or knee |
| 2 | Extensive movement of one joint or extensive movement of one joint and slight movement of one other joint |
| 3 | Extensive movement of two joints |
| 4 | Slight movement of all three joints of the HL |
| 5 | Slight movement of two joints and extensive movement of the third |
| 6 | Extensive movement of two joints and slight movement of the third |
| 7 | Extensive movement of all three joints of the HL |
| 8 | Sweeping with no weight support or plantar placement of the paw with no weight support |
| 9 | Plantar placement of the paw with weight support in stance only (i.e., when stationary) or occasional, frequent, or consistent weight-supported dorsal stepping and no plantar stepping |
| 10 | Occasional weight-supported plantar steps; no FL–HL coordination |
| 11 | Frequent to consistent weight-supported plantar steps and no FL–HL coordination |
| 12 | Frequent to consistent weight-supported plantar steps and occasional FL–HL coordination |
| 13 | Frequent to consistent weight-supported plantar steps and frequent FL–HL coordination |
| 14 | Consistent weight-supported plantar steps, consistent FL–HL coordination, and predominant paw position during locomotion is rotated (internally or externally) when it makes initial contact with the surface as well as just before it is lifted off at the end of stance; or frequent plantar stepping, consistent FL–HL coordination, and occasional dorsal stepping |
| 15 | Consistent plantar stepping and consistent FL–HL coordination and no toe clearance or occasional toe clearance during forward limb advancement; predominant paw position is parallel to the body at initial contact |
| 16 | Consistent plantar stepping and consistent FL–HL coordination during gait and toe clearance occurs frequently during forward limb advancement; predominant paw position is parallel at initial contact and rotated at lift off |
| 17 | Consistent plantar stepping and consistent FL–HL coordination during gait and toe clearance occurs frequently during forward limb advancement; predominant paw position is parallel at initial contact and lift off |
| 18 | Consistent plantar stepping and consistent FL–HL coordination during gait and toe clearance occurs consistently during forward limb advancement; predominant paw position is parallel at initial contact and rotated at lift off |
| 19 | Consistent plantar stepping and consistent FL–HL coordination during gait, toe clearance occurs consistently during forward limb advancement, predominant paw position is parallel at initial contact and lift off, and tail is down part or all of the time |
| 20 | Consistent plantar stepping and consistent coordinated gait, consistent toe clearance, predominant paw position is parallel at initial contact and lift off, and trunk instability; tail consistently up |
| 21 | Consistent plantar stepping and coordinated gait, consistent toe clearance, predominant paw position is parallel throughout stance, and consistent trunk stability; tail consistently up |

Cite from: Basso DM, Beattie MS, Bresnahan JC. Graded histological and locomotor outcomes after spinal cord contusion using the NYU weight-drop device versus transection. Experimental neurology 1996; 139(2): 244-256.
